# Minimum requirements and optimal testing strategies of a diagnostic test for leprosy as a tool towards zero transmission: A modeling study

**DOI:** 10.1371/journal.pntd.0006529

**Published:** 2018-05-25

**Authors:** David J. Blok, Sake J. de Vlas, Annemieke Geluk, Jan Hendrik Richardus

**Affiliations:** 1 Department of Public Health, Erasmus MC, University Medical Center Rotterdam, Rotterdam, The Netherlands; 2 Department of Infectious Diseases, Leiden University Medical Center, Leiden, The Netherlands; Beijing Institute of Microbiology and Epidemiology, CHINA

## Abstract

**Background:**

The availability of a diagnostic test to detect subclinical leprosy cases is crucial to interrupt the transmission of *M*. *leprae*. In this study we assessed the minimum sensitivity level of such a (hypothetical) diagnostic test and the optimal testing strategy in order to effectively reduce the new case detection rate (NCDR) of leprosy.

**Methods and findings:**

We used the individual-based model SIMCOLEP, and based it on previous quantification using COLEP data, a cohort study of leprosy cases in Bangladesh. The baseline consisted of treatment with Multidrug therapy of clinically diagnosed leprosy cases, passive case detection and household contact tracing. We examined the use of a leprosy diagnostic test for subclinical leprosy in four strategies: testing in 1) household contacts, 2) household contacts with a 3-year follow-up, 3) a population survey with coverage 50%, and 4) a population survey (100%). For each strategy, we varied the test sensitivity between 50% and 100%. All analyses were conducted for a high, medium, and low (i.e. 25, 5 and 1 per 100,000) endemic setting over a period of 50 years.

In all strategies, the use of a diagnostic test further reduces the NCDR of leprosy compared to the no test strategy. A substantial reduction could already be achieved at a test sensitivity as low as 50%. In a high endemic setting, a NCDR of 10 per 100,000 could be reached within 8–10 years in household contact testing, and 2–6 years in a population testing. Testing in a population survey could also yield the highest number of prevented new cases, but requires a large number needed to test and treat. In contrast, household contact testing has a smaller impact on the NCDR but requires a substantially lower number needed to test and treat.

**Conclusions:**

A diagnostic test for subclinical leprosy with a sensitivity of at least 50% could substantially reduce *M*. *leprae* transmission. To effectively reduce NCDR in the short run, a population survey is preferred over household contact tracing. However, this is only favorable in high endemic settings.

## Author summary

The annual number of new leprosy cases has been stable in the past decade, indicating that transmission has not been yet been interrupted. As current control seems to be insufficient to bring down the number of cases, there is a need for novel tools to interrupt transmission. A diagnostic that permitted diagnosis of subclinical cases will likely be fundamental to achieve elimination and ultimately eradication. In this study we assessed the minimum sensitivity level of such a (hypothetical) diagnostic test and the optimal testing strategy in order to effectively reduce the new case detection rate (NCDR) of leprosy. We showed that a diagnostic test for subclinical leprosy could substantially reduce the NCDR in a high, medium and low endemic population. A significant impact could already be achieved at a test sensitivity level of 50%. To effectively reduce the NCDR in the short run, a population survey is preferred over household contact tracing. However, this is only favorable in high endemic settings, as in medium and low endemic settings testing in a population survey requires many more people to be tested and treated to prevent one new leprosy case.

## Introduction

Leprosy is an infectious disease caused by *Mycobacterium leprae*, affecting the skin, peripheral nerves, the mucosa of the upper respiratory tract and the eyes [1]. The most likely route of transmission of *M*. *leprae* is via the aerosolic route [2]. Individuals, who have close and frequent contact to a patient with leprosy, in particular within households, have the highest risk of acquiring the infection and developing leprosy [3, 4]. Currently, the main strategy to control leprosy, as recommended by the World Health Organization (WHO), is early detection of cases and treatment with multidrug therapy (MDT) [5]. Leprosy is diagnosed by clinicians based on the clinical signs and symptoms, along with the use of slit-skin smears and biopsies to respectively detect the presence of acid-fast bacteria and determine type of leprosy histologically [6].

Although the prevalence of leprosy has dropped immensely in the last 30 years, worldwide still more than 200,000 new cases of leprosy are detected annually [5]. This number has remained fairly stable over the last decade, indicating that transmission has not yet been interrupted. Global elimination has been a target since 1991, and more recently the target for leprosy has been set to achieve zero transmission [7, 8]. However, it is clear that the current strategy is not sufficient to achieve the goals within a reasonable time frame [9, 10]. For this reason, alternative strategies should be considered. Previous modeling studies have shown that treating people during the subclinical stage of leprosy has a larger impact on the new case detection rate (NCDR) than early (clinical) diagnosis and treatment [11]. Therefore, interventions such as the provision of chemoprophylaxis (antibiotics) or immunoprophylaxis (vaccination) to contacts of leprosy cases could substantially further reduce the NCDR [12–14]. Nevertheless, these are not yet routinely available nor accepted. A more efficient approach would be the use of a diagnostic test that allows identification of *M*. *leprae* infected individuals who are at risk of developing leprosy and constitute the major source of transmission.

The identification and validation of new sensitive biomarkers for *M*. *leprae* infection and (subclinical) leprosy is currently investigated in several leprosy endemic areas [15–19]. Host immunity after *M*. *leprae* infection is determined by host genetics, leading to a complex immuno-pathological spectrum associated with either dominant cellular or humoral immunity. The immune-mediated pathological leprosy spectrum compels detection of *M*. *leprae* infection to be based on multiple, diverse biomarkers specific for cellular as well as humoral immunity [15, 19]. The use of serological proteomics can help to unravel the biological pathway in the immunomodulation of leprosy for diagnostic purposes [20]. In addition, pathogen-based approaches identifying the presence of *M*. *leprae* in skin smears of contacts and patients may offer additional tools for prophylactic targeting [21, 22]. The availability of a specific and robust diagnostic test to detect infected individuals lacking clinical symptoms, would not only be beneficial to identify and treat cases at early stages before irreversible damage occurs but may also be crucial for breaking the transmission chain. As the prevalence of leprosy decreases, leprosy health care has been integrated into general health care causing decreased clinical expertise for diagnosing leprosy, leading to extended delays of diagnosis and as a result maintenance or re-emergence of infection. Implementation in general health care of a user- and field-friendly diagnostic test specific for leprosy may accommodate for the lack of leprologists in the field.

In this study we aim to identify the minimum requirements of an as yet hypothetical diagnostic test for subclinical leprosy (i.e. identifying infected individuals who will progress to disease) and its potential impact on the NCDR. In order to predict the added value, non-linearity of infectious disease patterns in the transmission process needs to be taken into account. For this purpose, we will use the individual-based model SIMCOLEP, which models *M*. *leprae* transmission and control of leprosy in a population structured by households. The model has previously been used to estimate future NCDR trends in Bangladesh, India, Brazil, and Indonesia, and to test the impact of various interventions targeting household contacts [9–11, 23, 24]. We use SIMCOLEP to assess the impact of a diagnostic test on NCDR under various assumptions of sensitivity, ranging from 50 to 100%. Furthermore, we investigate the optimal strategy of using such a test: household contact testing or a population survey. As the impact of test strategies might be dependent on the endemicity level, all analysis were conducted in a high (25 per 100,000), medium (5 per 100,000) as well as low (1 per 100,000) endemic setting [25].

## Methods

### Modeling approach

We used the individual-based model SIMCOLEP that simulates the spread of *M*. *leprae* in a population structured in households. It models life-histories of individuals, which are born and placed into households. Over time, individuals can create their own household or move to another household after marriage, during adolescence or after becoming a widow(er). Deaths of individuals are determined by death rates at birth [23, 26].

In the model, *M*. *leprae* transmission occurs when a susceptible individual has contact with an infectious individual. In the model, susceptibility of an individual to leprosy is randomly assigned. We assumed that 20% of the population is susceptible, implying that 80% will not develop leprosy, although the proportion of susceptibles is likely to be lower [1]. This assumption was made because a previous modeling study showed that assuming 20% susceptibles provided the best fit and that results did not significantly differed from assuming 5% or 10% susceptibles [23]. Two transmission processes are modeled separately: transmission in the general population and within-household transmission. The latter can be regarded as an additional probability of acquiring the infection if household contacts are infected. Infectivity is determined by the product of the contact rate, both in the general population and within households, and the probability of infection during a contact. An infected individual develops either paucibacillary (PB) or multibacillary (MB) leprosy, which is randomly determined based on the distribution of the type of leprosy. We assumed that only MB leprosy is infectious. After infection, an individual enters the asymptomatic state, which on average lasts 4.2 years for PB and 11.1 years for MB. Afterwards the individual proceeds to the symptomatic state, which lasts on average 5 years for PB leprosy after which self-healing occurs. An MB leprosy case remains symptomatic until treatment or death. The natural history of leprosy is modeled following Meima et al. [27]. The model also replicates control measures including treatment with multidrug therapy (MDT), passive case detection, and household contact tracing. All detected cases receive MDT treatment, and will not be infectious from then on. Relapses occurred with a rate of 0.001 per year. 90% relapses to MB and 10% to PB. A full description of the model can be found in Fischer et al. 2010 and Blok et al. 2015 [9, 23].

### Model quantification

We used previously published quantifications of the model based on the population and leprosy epidemiology in Nilphamari and Rangpur districts in Bangladesh. A full description of the data used to parameterize the birth, household movements and deaths of individuals, and leprosy epidemiology can be found in Fischer *et al*. 2010 [23].

Transmission of *M*. *leprae* was previously calibrated using data from the COLEP study [4]. The COLEP study population consisted of contacts of 1037 consecutively found new patients with leprosy in Nilphamari and Rangpur districts in Bangladesh. The data contain information about the genetic distance (i.e. kinship) of almost 22,000 contacts and were used to fit the prevalence of cases among contacts of different household sizes, the prevalence of cases among different types of relatives in 2003. Quantifications were not updated to more recent numbers, because a trial with chemoprophylaxis started afterwards. Our main focus was to estimate the impact of a diagnostic test for detection and subsequent effective treatment of *M*. *leprae* infected individuals progressing to disease, in addition to the mainstay control program, as recommended by the WHO [25]. The transmission contact rate in the general population and within household was set to 1.33 and 0.98, respectively [23]. The baseline NCDR of Nilphamari and Rangpur was 27 per 100,000 and the MB/PB ratio 20/80 in 2003 [4].

The leprosy control program in the modeled area was well-organized [28]. It consisted of passive case detection with an annual detection delay of on average of 2 years (standard deviation 1.4 years) [29] and continuous household contact tracing with a coverage of 90% [23]. During contact tracing only clinical leprosy cases can be diagnosed. We further included the protective effect of BCG vaccination prior to the infection, which was set to 60% [13]. In order to reflect a high, medium and low endemic setting, we ran the model given the current control until it reached a NCDR level of 25 per 100,000, 5 per 100,000 and 1 per 100,000. This was obtained after 2, 18 and 40 years, respectively.

### Scenarios

In different scenarios, we evaluated the impact of using a diagnostic test for subclinical leprosy on the NCDR trend under various sensitivity levels, ranging from 50% to 100%. We neglected lower levels of sensitivity, because it is not expected that such a diagnostic test will come on the market. Individuals testing positive will be treated with MDT [30].

We evaluated four testing strategies with a diagnostic test for subclinical leprosy in addition to the current leprosy control:

1. *Testing in household contacts of index patients without follow-up*. All household contacts of an index case were traced, clinically examined and tested with the diagnostic test for subclinical leprosy. Household contact testing was rolled out as a continuing routine program.
2. *Testing in household contacts of index patients with a 3-year follow-up*. All household contacts of an index patient were traced, clinically examined and tested using the diagnostic test for subclinical leprosy. All contacts were followed-up for a period of 3 years. During follow-up, all household contacts that were not positive before are clinically examined and tested again (once every follow-up). This testing strategy was rolled out as a continuing routine program.
3. *Testing in a population survey with a coverage of 50%*. Half of the population is clinically examined and tested during a one-time population survey.
4. *Testing in a population survey with a coverage of 100%*. The whole population is clinically examined tested during a one-time population survey.

All scenarios were compared to the no-testing strategy which only consisted of the current leprosy control program. Leprosy cases under treatment were excluded from testing. Testing compliance in individuals was assumed to be 100%. All strategies were evaluated in terms of its impact on the NCDR and its benefit and costs. The benefit of a testing strategy was measured by the number of prevented new leprosy cases calculated as the difference between the new cases (detected and undetected) of a testing strategy and the no-testing strategy. The costs of a testing strategy were measured in terms of number needed to test and treat. As tests that are not completely predictive may produce false positives, we assessed the number needed to treat given a test specificity of 100%, 95% and 85%. The impact on NCDR, and the benefits and costs of all strategies were evaluated in a high, medium and low endemic setting. The modeled time span was a 50-year period.

In a sensitivity analysis, we varied the passive case detection delay and MB/PB ratio. First, we increased the passive case detection delay to six years, representing an area with a less well-organized leprosy control program in place. Second, we increased the MB/PB ratio to 65/35, representing an area with relatively more MB than PB cases. The latter is relevant because we assume that only MB leprosy is contagious.

## Results

**Fig 1:**
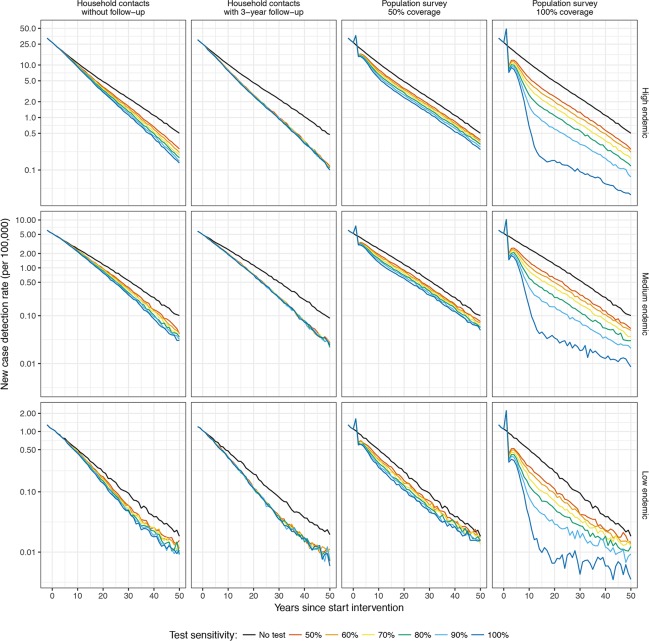
Impact of a diagnostic test used in household contacts and a population survey on the NCDR under various assumptions of sensitivity in a high, medium and low endemic setting. Four strategies were assessed: testing in 1) household contacts without follow-up, 2) household contacts with a 3-year follow-up, 3) a population survey with a coverage of 50%, and 4) a population survey with a coverage of 100%. Test sensitivities vary between 50% and 100%. The black line represents a strategy in which no diagnostic test was used (i.e. continuation of current control). High endemic is defined as 25 per 100,000 population, medium as 5 per 100,000, and low as 1 per 100,000.

Fig 1 presents the impact of a diagnostic test used in household contacts without follow-up and with a 3-year follow-up, and a one-time population survey with a coverage of 50% and 100% on the NCDR under various assumptions of test sensitivities. In all strategies, the use of a diagnostic test further reduces the NCDR of leprosy compared to the no-testing scenario. Household contact testing without follow-up decreases the NCDR gradually over time with slightly larger effects at higher levels of test sensitivities. If household contacts are additionally followed-up for 3 years, the impact of lower test sensitivities increases to the level of the highest test sensitivity. In a population survey of 50% and 100%, a substantial reduction can be seen in the short run followed by a gradual decline afterwards. The impact varies with sensitivity levels of the test: a higher sensitivity level of the test corresponds with a larger impact on the NCDR. In the short run, both population survey testing strategies result in a larger decrease in the NCDR than household contact testing. In a population survey with a coverage of 100%, the NCDR could be 40% lower within 10 years with a test sensitivity of at least 60% (See S1 Fig). In the long run, the impact of household contact testing on NCDR may be larger than that of a population survey, depending on the coverage and test sensitivity. In a high endemic setting, a NCDR of 10 per 100,000 could be reached within ten and eight years in household contact testing without and with follow-up, respectively. In a population survey testing strategy with a coverage of 50% and 100% this level could be reached within six and two years, respectively. The relative impact clearly varies with endemicity level with the high endemic setting showing the largest relative decrease, followed by medium and low endemic setting.

**Fig 2:**
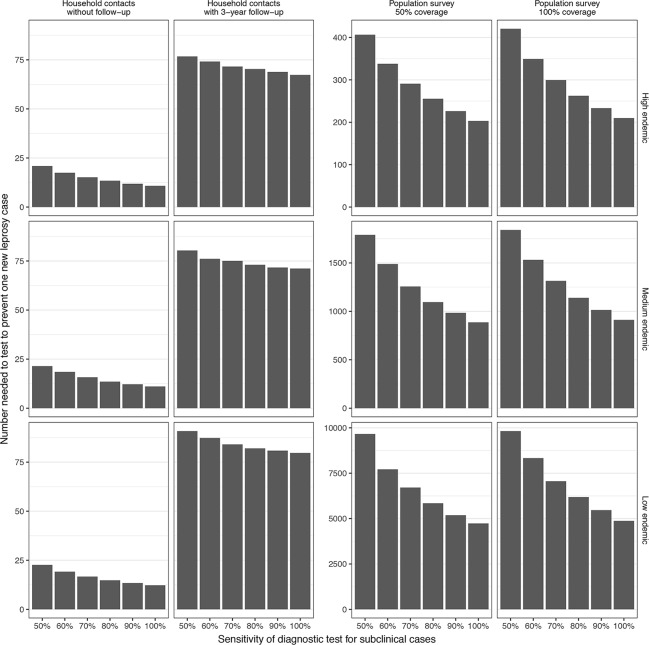
Number needed to test to prevent one new leprosy case. Results present the efficiency of a diagnostic test in household contacts and a population survey under various assumptions of sensitivity in a high, medium and low endemic setting. High endemic is defined as 25 per 100,000 population, medium as 5 per 100,000, and low as 1 per 100,000.

The number needed to test in order to prevent one new leprosy case decreases with level of sensitivity and increases with the endemicity level of the setting (see Fig 2). Household contact testing without and with follow-up require substantially fewer individuals to be tested to prevent one leprosy case compared to both population survey strategies. In a one-time population survey, testing in a high endemic setting requires the least number of people to be tested to prevent one new leprosy case. In a medium and low endemic setting this number is up to four and twenty-three times higher, respectively.

Table 1 summarizes the benefits and costs of all testing strategies with an assumed test sensitivity of 70% in a population of 1 million after 10 years. The numbers of prevented cases are approximately up to ten times higher in a population survey than in household contact testing. The highest number of prevented cases could be achieved in a high endemic setting. However, testing in a population survey would require substantially more people to be tested and treated than household contact testing. If the test is not completely specific, the number needed to treat further increases as a result of an increased number of false positives. This increase is much larger in a population survey than household contact testing, as more people are tested in a population survey. The costs of household contact testing decrease with the level of endemicity, whereas the costs of a population survey do not differ much across endemic settings.

**Table 1:** Number of prevented new leprosy cases and number needed to test and treat in a population of 1 million after 10 years using a test with 70% sensitivity.

Endemicity ^a^	Strategy	Prevented new leprosy cases	Number needed to test	Number needed to treat		
				100% ^b^	95% ^b^	85% ^b^
High	Household contact tracing without follow-up	200	10,700	160	690	1,700
	Household contact tracing with 3-year follow-up	500	83,400	300	4,400	12,700
	Population survey (50%)	990	479,800	390	24,400	72,300
	Population survey (100%)	1,910	959,700	780	48,700	144,700
Medium	Household contact tracing without follow-up	40	2,200	30	140	360
	Household contact tracing with 3-year follow-up	99	16,500	60	870	2,500
	Population survey (50%)	220	483,800	100	24,300	72,700
	Population survey (100%)	420	967,700	210	48,600	145,300
Low	Household contact tracing without follow-up	9	500	6	30	80
	Household contact tracing with 3-year follow-up	19	3,500	11	180	530
	Population survey (50%)	45	487,200	21	24,400	73,100
	Population survey (100%)	86	974,300	42	48,800	146,200

^a^ High is defined as 25 per 100,000 population, medium as 5 per 100,000, and low as 1 per 100,000)

^b^ Assumed test specificity: 100%, 95% and 85%

S1 Table provides the results of our sensitivity analysis. The number of prevented new leprosy cases is smaller in a setting with a less well-organized leprosy control program compared to a well-organized setting (i.e. Nilphamari and Rangpur districts in Bangladesh). In a setting with a high MB/PB ratio, more new leprosy cases can be prevented in a 10-year period. The number needed to test and treat is to a large extent comparable across all leprosy settings.

## Discussion

This paper assessed the impact of the use of a (hypothetical) diagnostic test for subclinical leprosy cases with a sensitivity that ranged between 50% and 100% on the NCDR in household contact testing and one-time population survey strategies. All strategies showed an additional reduction in the NCDR over time compared to the current control for all levels of sensitivity in a high, medium and low endemic setting. Testing in a population survey yields a higher impact on the NCDR in the short run compared to household contact tracing. In terms of prevented cases, a population survey is preferred over household contact testing. However, a population survey requires much more people to be tested and treated, especially in medium and low endemic setting, compared to household contact testing.

Our findings indicate that a test with a sensitivity as low as 50% could already result in a significant reduction of the NCDR. This suggests that to reduce transmission the availability of a diagnostic test for subclinical cases is more important than the level of sensitivity, which is very promising. Moreover, the impact of a test with a low test sensitivity could be increased by repeating the test in individuals testing negative. We showed that a test with a sensitivity of 50% used in a household contact testing strategy with a 3-year follow-up could reach a similar impact as a test with a sensitivity of 100%.

Testing in a population survey is most favorable to achieve short term reductions of NCDR in a high, medium and low endemic setting. The lower impact of household contact tracing compared to a population survey, especially in the short run, is primarily due to the limited exposure of the test that is confined to a household with an average size between 4 to 5 persons. A population survey would also result in a higher number of prevented new leprosy cases. However, it requires testing of many individuals and thereby commitment by the national leprosy control programs. Such an approach is only favorable and feasible in high endemic settings. Our results show that the numbers needed to test to prevent one new leprosy case in a medium and low endemic setting are approximately fourfold and twenty-threefold that of a high endemic setting, respectively.

Although tests specificity is of less importance if we aim to reduce transmission of *M*. *leprae*, it is relevant for determining the optimal testing strategy. If a test is not completely specific, the number needed to treat may increase dramatically. As the number of people tested is higher in a population survey than in household contact testing, it would also produce many more false positives (See Table 1). A solution to reduce the number of false positive is to apply a two-step approach, whereby a second test with high specificity is added [30].

Our study also highlights that the target of zero transmission might be difficult to achieve, even with a well-organized control program and the use of a diagnostic test for subclinical cases. Over a 50-year period, our model predicted that the NCDR in the high, medium and low endemic setting would be at most reduced to very low levels: 0.10–0.50, 0.01–0.10, and 0.005–0.025 per 100,000 in a high, medium and low endemic setting, respectively (Fig 1). This is mainly the result of the combination of a relative long incubation period of leprosy and detection delay. Areas that reach very low levels of NCDR require continuous monitoring for many more years to prevent maintenance or even re-emergence of *M*. *leprae*.

In this study we did not consider the impact of a test for asymptomatic infection for future NCDR, because such a test is only relevant if we assume that all infected individuals are infectious (i.e. can infect other people). In our model, we assumed that only infected individuals who progress to MB leprosy are infectious, implying those who progress to PB leprosy and those who do not develop leprosy do not contribute to the transmission. This assumption was made because it is still poorly understood whether and to which extent asymptomatic infected are infectious.

This study used previously published quantifications of the model based on the population and leprosy epidemiology in Nilphamari and Rangpur districts in Bangladesh. The advantage of using this quantification is that it was based on the COLEP study, which includes a large amount of very detailed information on this population, including data on the prevalence of cases among contacts of different household sizes and the prevalence of cases among different types of relatives [4]. The downside is that quantifications are based on 2003 data, which does not truly reflect current leprosy epidemiology. However, for the purpose of assessing potential impact of a diagnostic test this is less of an issue.

The primary concern of this study is about the extent to which our results are generalizable to other regions or countries with leprosy in the world. The leprosy control program in the Nilphamari and Rangpur districts of Bangladesh is more extensive than usual. The relative short detection delay of two years and active household contact tracing with a coverage of 90% is not common in leprosy control programs in other regions or countries. For that reason, we conducted a sensitivity analysis in which we increased the passive case detection delay to six years. Results show that fewer leprosy cases were prevented (S1 Table). The qualitative findings of our main results (the importance of sensitivity and the impact and efficiency of household contact tracing versus population survey) did not differ when implemented in a setting with a less well-organized control program.

Another concern with respect to generalizability is the distribution of the MB/PB leprosy. In Bangladesh the MB/PB ratio is approximately 20/80. Since we assumed in our model that only MB cases are infectious, the impact of a diagnostic test might differ in areas with strikingly different MB/PB ratios, such as in Brazil (65/35) or Indonesia (80/20) [5]. In a sensitivity analysis, we showed that in a setting with a MB/PB ratio of 65/35, the benefit of a diagnostic test is larger compared to our main results (S1 Table). This is because of the earlier detection of relative more MB cases.

Finally, this study did not look into combining the use of a diagnostic test for subclinical leprosy with additional novel strategies, such as a chemoprophylaxis, as this is beyond the scope of this study. Earlier modeling studies have shown that providing contacts with a single-dose of rifampicin (SDR) chemoprophylaxis can reduce the transmission of leprosy over time [11, 24]. It can be expected that adding chemoprophylaxis to our testing strategies would further reduce the transmission of leprosy, especially if the test sensitivity is not optimal. In that case, the contacts that were false negative might benefit from SDR.

## Conclusions

We showed that a diagnostic test for subclinical leprosy could substantially reduce transmission in a high, medium and low endemic population. The test sensitivity influences the impact on transmission in a population survey, but even with levels as low as 50% a substantial reduction could be achieved. To effectively reduce the NCDR in the short run, a population survey is preferred over household contact tracing. However, this is only favorable in high endemic settings, as in medium and low endemic settings testing in a population survey requires many more people to be tested and treated to prevent one new leprosy case.

## Supporting information

S1 Fig Relative reduction of the new case detection rate (NCDR) of various testing strategies compared to a strategy without testing (i.e. current control). Results present the relative impact of testing in household contacts without follow-up, with a 3-year follow-up, and in a population survey with a coverage of 50% and 100% under various assumptions of test sensitivity in a high, medium and low endemic setting. High endemic is defined as 25 per 100,000 population, medium as 5 per 100,000, and low as 1 per 100,000. The relative impact on the NCDR is calculated by dividing the NCDR of a testing strategy by the NCDR of the no testing strategy.
(TIF)

S1 Table Sensitivity analysis: A comparison of the benefit and cost of testing strategies in three leprosy control settings. Results present the number of prevented new leprosy cases, number needed to test and treat in three endemicity settings with a population of 1 million after 10 years using a test with 70% sensitivity. We compared the leprosy control setting of our main analysis to an area with a less-well organized leprosy control program in place (passive case detection delay of 6 years) and to a setting with relatively more MB than PB cases (MB/PB ratio: 65/35).
(DOCX)
