# Supplementary figures and images for: Minimum requirements and optimal testing strategies of a diagnostic test for leprosy as a tool towards zero transmission: A modeling study
Source: PLoS Negl Trop Dis. 2018 May 25;12(5):e0006529. doi: 10.1371/journal.pntd.0006529 (PMC5991769; doi:10.1371/journal.pntd.0006529)

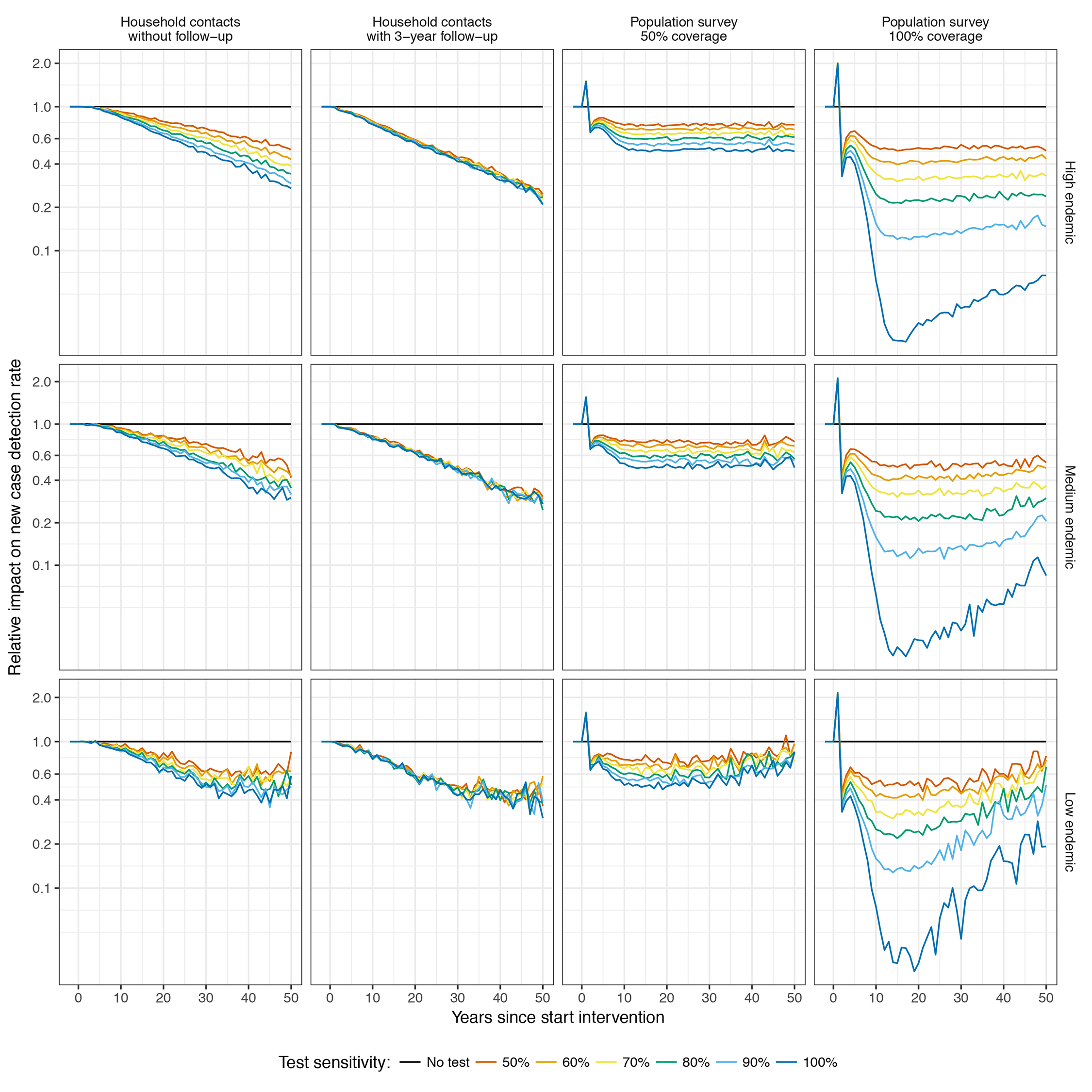

Supplement: S1 Fig — Results present the relative impact of testing in household contacts without follow-up, with a 3-year follow-up, and in a population survey with a coverage of 50% and 100% under various assumptions of test sensitivity in a high, medium and low endemic setting. High endemic is defined as 25 per 100,000 population, medium as 5 per 100,000, and low as 1 per 100,000. The relative impact on the NCDR is calculated by dividing the NCDR of a testing strategy by the NCDR of the no testing strategy. (TIF) [file pntd.0006529.s001.tif]
