# Supplementary material for: Minimum requirements and optimal testing strategies of a diagnostic test for leprosy as a tool towards zero transmission: A modeling study
Source: PLoS Negl Trop Dis. 2018 May 25;12(5):e0006529. doi: 10.1371/journal.pntd.0006529 (PMC5991769; doi:10.1371/journal.pntd.0006529)
Supplement: S1 Table — Results present the number of prevented new leprosy cases, number needed to test and treat in three endemicity settings with a population of 1 million after 10 years using a test with 70% sensitivity. We compared the leprosy control setting of our main analysis to an area with a less-well organized leprosy control program in place (passive case detection delay of 6 years) and to a setting with relatively more MB than PB cases (MB/PB ratio: 65/35). (DOCX) [file pntd.0006529.s002.docx]

S1 Table. Sensitivity analysis: a comparison of the benefit and cost of testing strategies in three leprosy control settings. Results present the number of prevented new leprosy cases, number needed to test and treat in three endemicity settings with a population of 1 million after 10 years using a test with 70% sensitivity. We compared the leprosy control setting of our main analysis to an area with a less-well organized leprosy control program in place (passive case detection delay of 6 years) and to a setting with relatively more MB than PB cases (MB/PB ratio: 65/35).

| Endemicity ^a^ | Strategy | Setting |  |  |  |  |  |  |  |  |
| --- | --- | --- | --- | --- | --- | --- | --- | --- | --- | --- |
|  |  | Bangladesh ^b^ | | | Less well-organized control program ^c^ | | | Reversed MB/PB ratio ^d^ | | |
|  |  | Prevented new leprosy cases | Number needed to test | Number needed to treat ^e^ | Prevented new leprosy cases | Number needed to test | Number needed to treat ^e^ | Prevented new leprosy cases | Number needed to test | Number needed to treat ^e^ |
| High | Household contact tracing without follow-up | 200 | 10,700 | 690 | 190 | 9,755 | 630 | 400 | 10,000 | 890 |
|  | Household contact tracing with 3-year follow-up | 500 | 83,400 | 4,400 | 450 | 75,300 | 4,000 | 970 | 64,400 | 3,800 |
|  | Population survey (50%) | 990 | 479,800 | 24,400 | 890 | 480,200 | 24,400 | 1,250 | 480,700 | 24,700 |
|  | Population survey (100%) | 1,910 | 959,700 | 48,700 | 1,740 | 960,300 | 48,700 | 2,390 | 961,200 | 49,300 |
| Medium | Household contact tracing without follow-up | 40 | 2,200 | 140 | 25 | 1,400 | 90 | 80 | 2,100 | 190 |
|  | Household contact tracing with 3-year follow-up | 100 | 16,500 | 870 | 60 | 10,700 | 560 | 175 | 13,000 | 770 |
|  | Population survey (50%) | 220 | 483,800 | 24,300 | 145 | 484,900 | 24,300 | 265 | 484,500 | 24,400 |
|  | Population survey (100%) | 425 | 967,700 | 48,600 | 280 | 970,000 | 48,600 | 510 | 969,100 | 48,800 |
| Low | Household contact tracing without follow-up | 9 | 500 | 30 | 4 | 200 | 15 | 17 | 400 | 40 |
|  | Household contact tracing with 3-year follow-up | 19 | 3,500 | 180 | 9 | 1,500 | 90 | 36 | 2,600 | 160 |
|  | Population survey (50%) | 45 | 487,200 | 24,400 | 19 | 487,900 | 24,400 | 54 | 487,400 | 24,400 |
|  | Population survey (100%) | 86 | 974,300 | 48,800 | 37 | 975,800 | 48,800 | 103 | 974,900 | 48,800 |

^a^ High is defined as 25 per 100,000 population, medium as 5 per 100,000, and low as 1 per 100,000)

^b^ Detection delay 2 years; MB/PB ratio 20/80 (original setting)

^c^ Detection delay 6 years; MB/PB ratio: 20/80

^d^ Detection delay 2 years; MB/PB ratio: 65/35

^e^ 95% test specificity
